# Non-Pharmacological Interventions towards Preventing the Triad Osteoporosis-Falls Risk-Hip Fracture, in Population Older than 65. Scoping Review

**DOI:** 10.3390/jcm9082329

**Published:** 2020-07-22

**Authors:** Alba Peraza-Delgado, María Begoña Sánchez-Gómez, Juan Gómez-Salgado, Macarena Romero-Martín, Mercedes Novo-Muñoz, Gonzalo Duarte-Clíments

**Affiliations:** 1 University Hospital Nuestra Señora de Candelaria, 38010 Santa Cruz de Tenerife, Spain; alba.peraza.2a@gmail.com; 2 University School of Nursing, Candelaria N.S. University Hospital, University of La Laguna, 38010 Santa Cruz de Tenerife, Spain; begonasanchez@gmail.com (M.B.S.-G.); extgduartcl@ull.edu.es (G.D.-C.); 3 Department of Sociology, Social Work and Public Health, Faculty of Labour Sciences, University of Huelva, 21007 Huelva, Spain; 4 Safety and Health Postgraduate Program, Universidad Espíritu Santo, Guayaquil 092301, Ecuador; 5 Department of Nursing. University of Huelva, 21071 Huelva, Spain; 6 Nursing Department, Faculty of Health Sciences. University of La Laguna, 38200 Tenerife, Spain; mernov@ull.es

**Keywords:** osteoporosis, hip fractures, risk factors, primary prevention, accidental falls

## Abstract

Osteoporosis leads to increased risk of falls, and thus an increase in fractures, highlighting here hip fractures, that result in high mortality, functional disability, and high medical expenditure. The aim is to summarise the available evidence on effective non-pharmacological interventions to prevent the triad osteoporosis/falls risk/hip fracture. A scoping review was conducted consulting the Scientific Electronic Library Online (Scielo), National Institute for Health and Care Excellence (NICE), Cumulative Index to Nursing & Allied Health Literature (CINAHL) y PubMed.databases. Inclusion criteria were articles published between 2013 and 2019, in Spanish or English. In addition, publications on a population over 65 years of age covering non-pharmacological interventions aimed at hip fracture prevention for both institutionalised patients in long-stay health centres or hospitals, and patients cared for at home, both dependent and non-dependent, were included. Sixty-six articles were selected and 13 non-pharmacological interventions were identified according to the Nursing Interventions Classification taxonomy, aimed at preventing osteoporosis, falls, and hip fracture. The figures regarding the affected population according to the studies are alarming, reflecting the importance of preventing the triad osteoporosis, falls risk, and hip fracture among the population over 65 years of age. The most effective interventions were focused on increasing Bone Mineral Density through diet, exercise, and falls prevention. As a conclusion, primary prevention should be applied to the entire adult population, with special emphasis on people with osteoporosis.

## 1. Introduction

Osteoporosis is an asymptomatic skeletal disease characterised by low bone mass density and structural deterioration of the bone tissue, resulting in increased bone fragility and susceptibility to fractures, especially hip fractures. Currently, hip fractures are considered one of the most common causes of hospital admission to orthopaedic surgery and traumatology services, affecting more than 200 million people worldwide [1]. In addition, admittances amount to more stays than those caused by diabetes, ischemic heart disease, or breast cancer [2,3], being one of the diseases with greatest socioeconomic impact given its high morbidity and its impact on mortality [3,4,5].

The bone capital is constituted between 15–30 years of age, and in the 10 years following menopause, in the case of women, or around 50 for men, it is a preventive factor for hip fracture. As we grow, bone mass begins to decrease and bone porosity increases, making bones more fragile. This, in turn, increases the chances of suffering a hip fracture [6,7].

The World Health Organization (WHO), in joint action with the International Osteoporosis Foundation (FIO), states that “the number of hip fractures due to osteoporosis is expected to triple over the next 50 years from 1.7 million in 1990 to 6.3 million by 2050”. This means a 6% increase in its incidence among males and 21% in women over 65 years of age. It is important to note that three out of four cases will be women [7,8,9]. Hip fracture rates show great variability between countries, with around 90% of cases in people over 65 years of age. Considering demographic trends, an increase in the number of hip fractures is expected in the coming years [10].

The evolution of the incidence rate of hip fracture has not developed uniformly. However, in most countries, it has increased. Thus, in countries such as the United States, between 1928 and 1942 and from 1973 to 1982, the incidence of hip fracture has been five times higher, from 135.8 to 675.8 per 105 inhabitants. A similar case occurs between 1992 and 2005, where the annual mean of hip fractures was 957.3 per 105 inhabitants in women, and 414.4 per 105 inhabitants in males [11]. These figures are close to those from other countries such as Australia, where there was an increase of approximately 11% in both sexes between 1997 and 2007, from 14,909 to 16,534 cases, estimating that the number of hip fractures will have increased between four and five times by the year 2051 [12].

In Spain, the prevalence of falls among the population over 65 years who reside in the community is around 32%, and between 45–49% among institutionalised people. Falls increase proportionally with age in both sexes and in all ethnic groups. A total of 119,857 hip fractures are recorded among males, and 415.421 in females. The incidence rates per sex were 259.24/105 inhabitants/year in males, and 664.79/105 inhabitants/year in women in 1997; 325.30/105 inhabitants/year and 766.37/105 inhabitants/year in 2010 for males and females, respectively [1,10,13].

Given the relevance of the problem, it is important to get acquainted with the modifiable risk factors, and those that are not, related to osteoporosis, falls risks, and hip fracture. Unmodifiable risk factors include [14]:

- Age: In general, for every decade, the risk of fracture is multiplied by 1.4–1.8.
- Sex: Women have three times more fractures than men.
- Ethnicity: Caucasians have a higher risk of fracture.
- Family history of fracture.
- Early menopause.

Modifiable risk factors include [15,16,17,18,19,20,21,22,23,24,25,26]:

- Body Mass Index (BMI).
- Toxic habits: tobacco and alcohol.
- Diet.
- Sedentary lifestyle and/or low mobility.
- Diseases and medicines.
- Environment (obstacles at home, in the person’s environment...).
- Fear of falling.

Of all of the above, a personal history of fracture in adulthood, low weight, smoking habit, and corticosteroid use generates greater risk. In particular, malnutrition is associated with increased predisposition towards hip fractures. However, higher fat and lean mass are associated with reduced rates of hip fracture. Also, vision problems, early menopause (before age 45), dementia, weak health, low calcium and vitamin D intake, low physical activity, and alcohol consumption (>2 units per day) generate less risk [1,14,17,18,19,20,21,22,23,24,25,26].

This is why it is important to assess the risk of hip fracture. Taking into account the most important risk factors, different evaluation scales have been developed. The tool developed by WHO from studies of population groups in Europe, North America, Asia and Australia is called FRAX ^®^ (Bone fragility fracture risk calculator) [University of Sheffield, Sheffield, UK] [27]. The systematic use of this tool plays a relevant role in Primary Care (AP) and it is based on individual models that use clinical risk factors with the bone mineral density (BMD) of the hip, allowing the calculation of the 10-year probability of fracture, both of the hip and other osteoporotic fractures such as clinical vertebral, forearm or shoulder fractures. [27,28].

From the point of view of primary and secondary prevention, it is important to consider that osteoporosis, falls, and hip fracture are not isolated phenomena, but are part of the same chain of risk. Thus, osteoporosis coupled with modifiable factors create a risk of falls. This one, in turn, causes the hip fracture risk to increase.

Therefore, this work aims to answer the question “What are the most effective non-pharmacological interventions to prevent the triad: osteoporosis, falls risk, and hip fracture in the population over 65 years?”, so as to identify the available evidence on effective non-pharmacological interventions to prevent this triad.

## 2. Experimental Section

The literature was reviewed following the Arksey and O’Malley’s [29] scoping review method. The Preferred. Reporting Items for Systematic Reviews and Meta-Analyses (PRISMA) [30] international standard for revisions was applied afterwards in order to collect articles data by following a logical structure and to increase quality, integrity, and consistency, as well as having greater transparency of the revisions and improving their interpretation. The following question was proposed: What are the most effective non-pharmacological interventions to prevent the triad: osteoporosis, falls risk, and hip fracture in the population over 65 years of age? According to the PICO (Patient or problem, Intervention, Comparison, Outcome) strategy [31], the items Population (population over 65 years of age); Intervention (preventive measures); Comparison (not preventive measures), and Outcome (osteoporosis, falls risk, and hip fracture) were stablished.

The following electronic databases were consulted: Scielo, National Institute for Health and Care Excellence (NICE), Cumulative Index to Nursing & Allied Health Literature (CINAHL), and PubMed. The key words selected were: aged; frail elderly; hip fractures; prevention and control; primary prevention; primary care; nurses; smoking; body fat mass; food; nutrition; diet; exercise; exercise therapy; motor activity; treatment; risk factors; accidental falls; osteoporosis; bone mineral density; incidence; epidemiology. They were combined using the booleans AND and OR resulting in the search strategies summarised in Table 1. The search was conducted between November and December 2019.

As eligibility criteria, the search was limited to: published between 2013 and 2019 in Spanish and English, and with access to the full text. Including articles with a population over the age of 65 and covering non-pharmacological interventions aimed at the prevention of hip fracture, both for institutionalised patients in long-stay health centres or hospitals, and patients cared for at home, both dependent and non-dependent. Also, risk factors that increase the likelihood of osteoporosis, falls, and hip fracture such as diet, toxic habits (tobacco, caffeine, alcohol), underlying diseases, medication, activity and exercise, and multifactorial programmes were assessed. No specific ethnicity was selected. This was complemented by a manual search in relevant websites such as WHO, International Osteoporosis Foundation (FIO), Spanish Society of Rheumatology, and Spanish Society of Internal Medicine.

The references were first reviewed by title and summary for pre-screening. Those studies that met the eligibility criteria were selected. The full critical reading assessment was performed by two researchers, and the disagreements were resolved by a third evaluator.

Critical appraisal was performed following the Critical Appraisal Skills Programme (CASP) [32], the Berra S. et al. tool for descriptive studies [33], and Appraisal of Guidelines Research and Evaluation (AGREE) for clinical practice guidelines [34]. Those who met the CASP screening requirements and scored more than five on the total of the used grid were selected. The structure of the Scottish Intercollegiate Guidelines Network (SING) [35] was used for the evidence synthesis.

The relevant data were extracted from each article, and a table of synthesis of results was prepared, including authorship, method, and summary of results. The extraction was carried out by pairs and, whenever there was some mismatch, it was subjected to the assessment of a third evaluator. Data were gathered by type of intervention:

- Lifestyle modifications: diet, and activity and physical exercise.
- Falls prevention: medication, balance-enhancing therapies, use of incontinence absorbers, and multifactorial programmes.
- Other measures: use of hip protectors.

Interventions aimed at preventing osteoporosis, falls, and hip fracture were identified and classified according to the Nursing Interventions Classification (NIC) taxonomy: 1100 nutrition management; 6490 falls prevention; 2380 medication management; 1850 improving sleep; 486 environmental management: safety; 5510 health education; 1806 help with self-care: transfer; 0221 exercise therapy: ambulation; 0200 exercise promotion; 4978 improve communication: visual deficit; 4720 cognitive stimulation; 6460 dementia management; and 0610 urinary incontinence care. The results were structured by meeting the identified interventions.

As for the information bias of the individual studies, it is specified in the synthesis. To avoid the risks of bias between overrepresented studies, the selected individual studies also included in the selected reviews were identified and eliminated. This prevented biases that affect cumulative evidence.

## 3. Results

**Figure 1:**
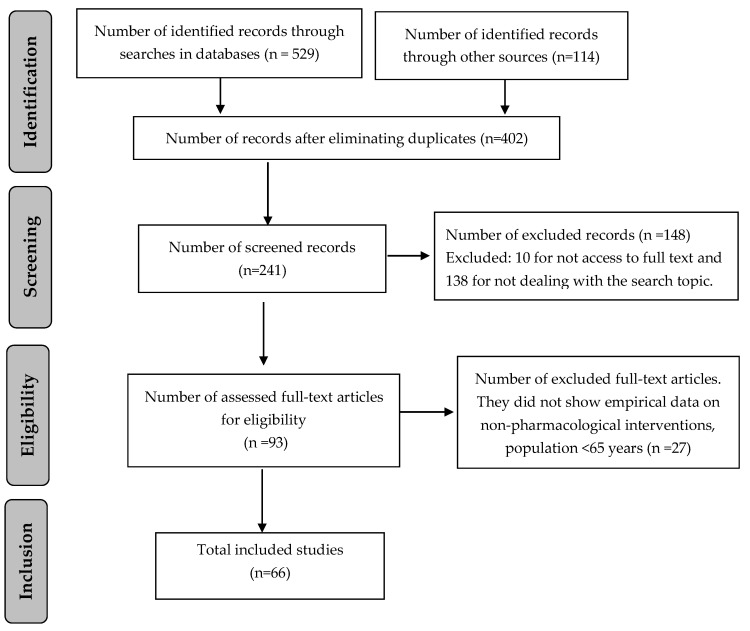
Flow chart of screening process.

A total of 643 references were identified, from which 66 were selected according to eligibility criteria as presented in Figure 1.

The results section may be divided by subheadings. It should provide a concise and precise description of the experimental results, their interpretation, as well as the experimental conclusions that can be drawn.

### 3.1. Lifestyle Modifications to Prevent Osteoporosis and Hip Fracture

#### 3.1.1. Diet

Calcium intake decreases bone loss, though it does not have a direct effect on the reduction of fractures in postmenopausal patients with osteoporosis. The recommended amount for patients with osteoporosis or under glucocorticoid treatment is 1500mg/day. That is, between four and four servings are needed to meet calcium needs, considering that a serving of dairy is equivalent to about 300 mg of this mineral (250 mL of milk = 2 yoghurts = 30 g, or 2 slices of cured cheese = 100 g of blue fish eaten with thorns such as 4 sardines in oil = 188 g of dried figs = 200 g of raw chickpeas = 286 g of tofu= 330 g of spinach or chard) [36,37,38].

About 50% of the population with osteoporosis has low serum levels of vitamin D, which is essential for bone development and maintenance as it helps the calcium absorption from food in the bowel, and also ensures the correct renewal and mineralisation of bone tissue [1,39,40,41].

This deficiency could be caused by several reasons such as insufficient intake, limited exposure to sunlight, or because the kidneys cannot convert vitamin D into its active form in the body. In adults, vitamin D deficiency causes osteomalacia, which causes bone pain and muscle weakness [1,7,39,40,41,42,43]. Foods containing this vitamin are quite limited: fatty fish (salmon, tuna, sardines, and mackerel); eggs; liver; and, in some countries, fortified foods such as margarine, dairy, and cereals such as in the United States, where the milk supply is fortified with 400 IU of vitamin D per litre, as well as many plant-based alternatives, such as soy milk, almond milk, and oat milk [7,44,45,46].

Vitamin D is acquired mainly through sun exposure, but this should be moderate. For example, in summer, short exposures (15–20 min) are advised outside of peak solar radiation hours; in autumn and winter, exposures should be increased [39,42]. In people with skin pathologies, brief exposures are recommended. This exhibition should be repeated two to three times per week [43]. Still, in published studies, sun exposure alone does not yield positive results on hip fractures, but vitamin D (800 IU/day) and calcium supplementation is recommended in institutionalised elderly patients [44,45,46].

As for the intake of isoflavones, nutritional supplements (magnesium, copper, zinc, iron, silica, boron, strontium, and manganese), and the consumption of proteins, their effectiveness in terms of decreased hip fracture is not so clear; there is little scientific evidence in this regard [37,47,48,49,50,51].

#### 3.1.2. Tobacco, Alcohol, and Caffeine

Alcohol, caffeine, and tobacco consumption should be avoided, as they increase the risk of frailty fracture by demineralising the bone [18]. The maximum recommended consumption is 15 packs of tobacco/year, 3 units of alcohol/day (1 unit: 8–10 g of alcohol), and 4 cups of coffee/day [52,53,54,55].

#### 3.1.3. Activity and Physical Exercise

Exercise should be performed on a regular basis and with the appropriate intensity for each person, assessed by considering their age, physical condition, and presence of diseases. For example: aquatic exercise is a viable and interesting strategy for older women with osteoporosis who have balance problems and difficulties in exercising on the ground [56]. High-intensity exercise has a significant effect on femoral neck Bone Mineral Density (BMD), but can also lead to problems in the lumbar spine in postmenopausal women, so caution should be exercised [57].

Carrying out muscle-strengthening activities and improving balance is recommended at least 3 days a week for people over 65 years, especially for those with mobility difficulties. Exercise against resistance (contractions of muscle groups with weights or bands) and maintenance exercise (walking, climbing stairs, cycling, swimming, doing Pilates…) help increase muscle strength, tolerance to exercise, and self-confidence [58]. Impact and gravity-based exercises are also effective as the bone adapts and responds to the main direction of mechanical loads (trabecular reorientation) such as shearing, compression, traction, bending, and torsion [58].

The use of oscillating or vibrating platforms for strength and balance exercises increases functional mobility more than exercises not performed on a platform [57,58].

No evidence has been found on the usefulness of Tai-Chi or unipodal exercises for fracture prevention, although they help maintain balance [58].

### 3.2. Falls Prevention

#### 3.2.1. Medication

It is advisable to remove or minimise all medicines in general. Special attention should be paid to drugs: psychoactives, those that decrease BMD, and which imply a risk of postural hypotension [43].

Drugs responsible for falls increase in older people:

o Psychoactives: hypnotics, anxiolytics, antidepressants, antiepileptics, benzodiazepines, opioids, etc. [59,60]

o Drugs at risk of postural hypotension: diuretics, antihypertensives [61,62,63,64]. Medication that can cause postural hypotension, foot problems and the use of appropriate footwear should also be evaluated [43,65,66].

Drugs that decrease BMD: glucocorticoids, l-thyroxine, heparin, antiepileptics, neuroleptics, chemotherapy, GnRH, aromatase inhibitors, methotrexate, cyclosporine A, vitamin A and synthetic retinoids, lithium, and antidepressants [54,67].

The recommended treatment is bisphosphonate. People with this treatment show a substantially lower risk of frailty fracture and mortality [68].

#### 3.2.2. Exercise Therapies to Improve Balance

Using the cane requires less attention while walking than when using walking aids, and its use may therefore prevent falls. However, the forward body inclination while walking with a cane may also favour them [45]. The use of canes improves confidence and functional capacity of the person, although these can as well limit the ability to obtain information from the environment and/or perform simultaneous tasks [7].

The cane improves balance in stroke patients and significantly reduces body sway in patients with peripheral vestibular balance disorders [68].

#### 3.2.3. Use of Incontinence Absorbers

Urgency, frequency, nocturia, and urinary incontinence are associated with a higher likelihood of falls among the elderly [7,69], as they often lead to people having to get up several times at night to urinate or forces them to use incontinence absorbers, thus limiting hip joint movement and walking skills, and increasing the risk of falling and, therefore, of fractures [25,66].

#### 3.2.4. Multifactorial Intervention Programmes

Multifactorial programmes focused on the systematic assessment of different risk factors and individualised intervention are highly useful in reducing the occurrence of falls among older people. Multifactorial intervention programmes (both in the community and in institutionalised populations) that have shown to be effective in preventing falls include the following elements: regular physical exercise to gain muscle strength and balance, advice instructions and interventions on risks at home, assessment and management of vision, and review of pharmacological treatments (modification or decrease) [20].

When promoting the participation of the elderly in multifactorial intervention programmes to prevent falls, it is important that health professionals assess the possible barriers for their implementation (fear of falling, physical barriers...) [21]. In addition, a careful approach should be followed as falls prevention programmes may as well be ineffective or have adverse effects [70,71].

In addition, it is also necessary to consider the user’s environment so as to adapt it and help the person with the necessary nursing interventions towards the promotion of their autonomy. It is also necessary to train both them and their relatives and/or caregivers in the correct handling of the environment, that is, training and learning on transfers (bed-armchair-WC), and modifications of the environment (having chairs with armrests, installing handles in the toilet and room, raising the toilet seat, evaluating the height of the bed, railings...) [71]. However, programmes based solely on changes in the environment and the education of patients and family members do not appear to be able to reduce, on their own, the risk of falls [27].

In the case of geriatric institutions, in order to reduce the incidence of falls, it is recommended to carry out individual education on risk factors and prevention strategies, as well as establishing targets. These strategies, conducted by a trained occupational therapist, reduce the frequency of falls in high-risk seniors living in the community [25,28,72].

### 3.3. Other Measures

#### Use of Hip Protectors

These protectors are placed in the hip area and their function is to absorb the impact of falls and reduce the risk of proximal femur fractures. Its use does not imply a decrease in the number of falls in at-risk patients and, although its effect on the reduction of fractures is not clear, its use is indeed recommended, mainly two-sided protectors in elderly people at high risk of fracture, especially institutionalised ones, as long as they agree to use them and these are properly applied [73].

As for their adverse effects, these are mild and rare and, therefore, do not limit their recommendation. However, many people refuse to use protectors due to the discomfort, difficulty in using them with absorbers, and the need to receive help to place them [73,74].

Results are summarised in Table 2, Table 3, Table 4 and Table 5.

As a summary of the results, Table 6 identifies the interventions described in the consulted literature and their correspondence with the NIC nurse interventions and the level of evidence and degree of recommendation classified with SING.

## 4. Discussion

In response to the research question, 13 scientifically evidenced non-pharmacological interventions aimed at the prevention of the triad osteoporosis, falls risks, hip fracture in a population over 65 years have been found.

The main recommendations extracted were:

- Evidence level 1+/Degree of recommendation C: Ca and vitamin D supplements in patients treated with glucocorticoids, that consume isoflavones, and with an intake of the following supplements: magnesium, copper, zinc, iron, silica, boron, strontium, manganese, and over-protein consumption [7,14,44,53,75].
- Evidence level 2++/Degree of recommendation B: Ensure a contribution of 1500 mg of calcium/day (each serving of dairy: 300 mg) and vitamin D, exposure to sunlight 15–20 min 2–3 days per week, exercise regularly and progressively (abandon sedentary lifestyle), abandon toxic habits or, if not possible, decrease them as much as possible (alcohol and tobacco), decrease caffeine consumption (increases urinary calcium excretion and hinders its absorption), exercises on vibrating platform [1,7,44,45,46,52,53,54,55,58].
- Evidence level 2+/Degree of recommendation B: Avoid falls risk factors, control medication and diseases, perform educational programmes, adapt the environment and home, address interventions in multifactorial and multidisciplinary programmes, use aid devices such as the cane, and be careful with the use of incontinence absorbers [70].
- Evidence level 2-/Degree of recommendation D: Use of hip protectors, consumption of tea, onion, or vitamin K79 supplements [73,74].

Thus, our study showed the importance of carrying out interventions to prevent the triad osteoporosis, falls risks, hip fracture such as: a calcium-rich diet of 1500mg/day to increase bone mass, in addition to a vitamin D-rich contribution that is acquired mainly through sun exposure, but moderately as recommended by the clinical practice guidelines for patients at risk of frailty fractures [36,37]. Toxic habits such as caffeine, alcohol, and tobacco should be avoided, or at least decrease their consumption [52,53,54,55]. Regarding the intake of isoflavones, nutritional supplements (magnesium, copper, zinc, iron, silica, boron, strontium, and manganese), and protein consumption, there is little scientific evidence to demonstrate their direct effectiveness in reducing hip fractures [6,37,47,48,49,50,51].

In addition, exercising promotes the mass, architecture, and bone structure adaptation, and reduces falls risk. This is especially so with those activities aimed at improving balance and muscle strengthening, thus preventing the triad: osteoporosis, falls risk, and hip fracture [52,53,54,55,56]. Among the activities that may be carried out, water exercises stand out, especially for those persons with balance problems and difficulties in exercising on the ground; also, exercises against resistance (muscle group contractions with weights or bands) and maintenance exercise (walking, climbing stairs, cycling, swimming, Pilates...) that help increase muscle strength, exercise tolerance and self-confidence [54,58].

Special emphasis should be placed on medication adjustment and control of underlying diseases as they increase the presence of osteoporosis and falls, causing an increased risk of hip fracture [61,62,63,64]. These are as psychoactive drugs (hypnotics, anxiolytics, antidepressants, antiepileptics, benzodiazepines, opioids, etc.), those that decrease bone mass density (glucocorticoids, l-thyroxine, heparin, antiepileptics, neuroleptics, chemotherapy, the Gonadotropin-releasing hormone (GnR), aromatase inhibitors, methotrexate, cyclosporine A, vitamin A, and synthetic retinoids, lithium, and antidepressants), and those that pose a risk of postural hypotension (diuretics, antihypertensives) [43,54,59,60,61,62,63,64,67].

Bisphosphonates are recommended as they have shown a substantially lower risk of frailty fracture and mortality among people who take them [68]. It is also important to adapt the environment to the needs of each person, to be careful and monitor episodes of incontinence, as well as the use of absorbents especially at night, and with regard to the use of hip protectors, this is limited, recommended only for those at high risk who accept their use and properly apply them [25,66,73,74].

The limitations of the study refer to the language restriction, the established time period, and the heterogeneity of studies on the incidence of hip fracture. The language of the articles included has been English and Spanish. However, this limitation is counteracted by the universality of both languages and the fact that most of the scientific information found is in either English or Spanish.

Another limitation is that only articles published between 2013–2019 were selected, and previous reference articles may exist. This is offset by the abundance of works existing in this period, by the inclusion of the quality criterion after the critical reading, and the reference search. Eventually, a total of 66 articles including randomised controlled trials, systematic reviews with or without meta-analysis, and clinical practice guidelines published between 2013–2019 were selected.

Another limitation is that data regarding the situation of the evolution of the hip fracture incidence is heterogeneous. The population represented is not entirely homogeneous, varying from one study to another, as well as the time period studied, the design of the works, the methods of analysis used, the results, and the conclusions drawn from each of them. Heterogeneity also makes quantitative synthesis difficult. Despite this, the figures in the studies remain alarming, reflecting the importance of preventing the triad osteoporosis, falls risk, and hip fracture among the population over 65 years. Authors should discuss the results and how they can be interpreted from the perspective of previous studies and of the working hypotheses. The findings and their implications should be discussed in the broadest context possible. Future research directions may also be highlighted.

## 5. Conclusions

Therefore, it is considered essential to establish a strategy for non-pharmacological interventions to prevent hip fracture due to fragility before and during pharmacological treatment in patients with osteoporosis or at risk of such fractures. These are interventions where the nurse has a primary job, but the responsibility is not only his/hers: It is essential to have professionals from other fields, be it healthcare or not (social workers, physiotherapists, occupational therapists...), in order to provide comprehensive care.

These therapies or actions should have a life cycle approach. Therefore, it is necessary to start from childhood in childcare consultations, emphasising the relevant measures to achieve a greater peak in bone mass such as adequate calcium intake, exercise, and non-acquisition of toxic habits. Subsequently, in adult consultations, educational interventions will aim to reinforce all these measures, in addition to those related to environment safety and the reduction of falls such as avoiding or decreasing the use of absorbers, as they limit mobility, thus increasing the risk of falling.

These interventions should include age-appropriate nutritional and health status assessments, as they contribute to better functional recovery and reduced mortality. For this purpose, a balanced diet is advised with an adequate intake of proteins and avoiding excess salt. The Mediterranean diet is fostered here for including foods that contain bioactive components with antioxidant, anti-inflammatory, and alkalising properties that contribute to the improvement of bone health. However, there are few specific studies that have assessed the effect of the Mediterranean diet on bone health.

On the other hand, given the evidence found and the high incidence of hip fractures, osteoporosis and falls among the population over 65 years, further research in non-pharmacological interventions is considered of particular relevance to prevent this triad and increase the level of evidence.

## Figures and Tables

**Table 1:** Search strategies, databases, and references.

Date	Database	Search Strategy	References Identified	Articles Included
27 August 2019	Pubmed	(“Hip fractures” AND (“preventi*” OR “control”) AND (“aged” OR “frail elderly”)	146	17
27 August 2019	Pubmed	“hip fractures”AND “nurs*” AND (“preventi*” OR “control”)	62	9
17 January 2019	Pubmed	(“Primary prevention” OR “Primary Care”) AND “hip fractures” AND (“aged” OR “frail elderly”)	32	4
29 January 2019	Pubmed	(“Smok*” AND “Body Fat Mass” AND “Bone Mineral Density”) AND “hip fractures”	1	1
31 January 2019	Pubmed	(“food” OR “nutrition” OR “diet”) AND (“hip fracture”) AND (“preventi*” OR “control”)	54	7
15 November 2019	Pubmed	(“Risk factors” AND “hip fractures” AND “treatment” AND “nurs*”)	7	1
18 November 2019	Scielo	“Osteoporosis” AND (“preventi*” OR “control”)	107	5
18 November 2019	Scielo	“Hip fractures” AND (“incidence” OR “epidemiology”)	24	4
18 November 2019	Scielo	Hip fractures AND (preventi* OR control)	44	2
28 August 2019	NICE (National Institute for Health and Care Excellence)	“Osteoporosis”	3	3
20 February 2019	Pubmed	(“fractura de cadera” AND (“prevención” OR “control”) AND (“Anciano*” OR “adulto mayor” OR “persona mayor” OR “anciano frágil” OR “anciano débil*” OR “anciano dependiente”)	6	1
20 February 2019	Pubmed	“fractura de cadera” AND (“factores de riesgo” OR “población de riesgo”)	1	0
21 February 2019	CINAHL	“fractura de cadera” AND (“factores de riesgo” OR “población de riesgo”)	1	1
21 August 2019	CINAHL	(“ejercicio” OR “terapia de ejercicios” OR “actividad física”) AND (“Anciano*” OR “adulto mayor” OR “persona mayor” OR “anciano frágil” OR “anciano débil*” OR “anciano dependiente”) AND “caídas”	4	0
21 February 2019	Scielo	Fractura de cadera AND osteoporosis	37	4

**Table 2:** Results from review articles.

Authors Year	Research Question	Method	Results	CASP ^1^	SIGN ^2^
Weaver CM, et al., 2016	Do calcium plus vitamin D supplements serve as an intervention for fracture risk reduction?	Meta-analysis	The calcium dosages ranged from 500 mg/day in one study to 1000–1200 mg/day in the remaining studies. Six studies provided 800 IU/day vitamin D, whereas dose levels were 400 and 700 IU/day in the remaining two studies. The use of calcium plus vitamin D supplements as an intervention for fracture risk reduction in both community-dwelling and institutionalised middle-aged to older adults.	6/10	1+/A
Santesso N, et al., 2014	Do hip protectors prevent hip fractures in older people?	Systematic review	The incidence of adverse events while wearing hip protectors, including skin irritation, ranged from 0% to 5%. Adherence, particularly in the long term, was poor. Hip protectors probably reduce the risk of hip fractures if made available to older people in nursing care or residential care settings, without increasing the frequency of falls. However, hip protectors may slightly increase the small risk of pelvic fractures. Poor acceptance and adherence by older people to hip protectors is a barrier to their use. Better understanding is needed of the personal and design factors that may influence acceptance and adherence.	8/10	1+/A
Fernandez MA, et al., 2015	How to manage hip fractures?	Systematic review	Prevention: Clinical assessment to identify medical causes for falling (e.g., postural hypotension, syncope, arrhythmia) should be coupled with basic investigations, such as a lying and standing blood pressure and a 12-lead ECG, and a review of current medications.	7/10	1+/C
Chen KW, et al., 2017	Is fragility a predictor of bone fractures?	Systematic review and Meta-analysis	The risk of fracture in the frail people was higher than that in both robust people (summary HR: 1.67; 95% CI [1.46–1.91]) and prefrail people (summary HR: 1.28; 95% CI [1.16–1.40], A subgroup analysis revealed that among female adults, older females with hip fracture, or those aged 65 years or more, those who were categorized as frail showed the highest fracture risk, followed by those who were categorized as prefrail.	8/10	1+/A
Rapp K, et al., 2018	Epidemiology of hip fracture and associated factors.	Systematic review	Hip fracture rates vary more than 100-fold between different countries. In most high-income countries, a rise in age-standardized hip fracture rates was observed until the 1980s and 1990s and a decrease thereafter. Risk factors: Diseases and drugs have been found to be associated with hip fractures and there is some evidence that fracture risk in later life is already programmed during foetal life and early childhood. Fifty percent occur in people with disability and in need of care. In nursing homes, approximately 4 fractures can be expected in 100 women per year. In people with intellectual or developmental disabilities, comparable risks of hip fracture occur 10–40 years earlier than in the general population.	7/10	1+/B
Romero Pérez A, et al., 2014	What are the effects of Mediterranean diet on bone health?	Systematic review	The Mediterranean diet is characterized by a high intake of olive oil, plant products, fish and seafood; a low intake of dairies, meat and meat products; and a moderate ethanol intake. These food items contain a complex array of naturally occurring bioactive molecules with antioxidant, anti-inflammatory and alkalinising properties that may contribute to the bone-sparing. Further large-scale studies are required to clarify the effect of Mediterranean diet on bone health, in order to establish the role of this diet in the prevention of osteoporosis.	7/10	1+/C
Poole CD, et al., 2014	Can cholecalciferol reduce the occurrence of hip fractures?	Meta-analysis	Prescribing cholecalciferol 800 IU daily to all adults aged 65 and over could reduce the number of hip fracture incidents from 65,400 to 45,700, saving almost 1700 associated deaths, whilst saving the UK taxpayer £22 million.	6/10	1+/B
Ballesteros-Álvaro AM, et al., 2013	Non-pharmacological interventions that are effective in preventing fragility hip fractures in Primary Care	Systematic review	The number of hip fractures due to osteoporosis is expected to triple over the next 50 years, from 1.7 million in 1990 to 6.3 million by 2050. This will mean an increase in the incidence of hip fractures in people in 65 years (three out of four will be women). The most important non-pharmacological interventions to prevent osteoporosis are those related to lifestyle changes (dietary modifications, exposure to sunlight, regular exercise practice, elimination of toxic habits such as alcohol and tobacco consumption, decrease in caffeine consumption, and exercises on the vibratory platform), to the prevention of falls (calcium and/or vitamin D intake, avoiding risk factors for falls, exercising, controlling medicines and diseases, educational programmes, interventions in the home and the environment, multifactorial intervention programmes, exercises on vibratory platform, use of cane, and use of incontinence absorbers), and also those related to the use of hip protectors.	7/10	1+/A
Tiedemann A, et al., 2013	Is physical activity important to prevent falls in the old age?	Literature review	Falls account for approximately 18% of emergency hospital admissions by older people. Studies undertaken in Sweden, the United States, and the United Kingdom have drawn attention to the significant direct health care costs required for the treatment of fall-related injury. A range of exercise programs that target balance and provide ongoing exercise are effective in preventing falls, for example, the Otago Programme of home-based balance and strength training and the LiFE program of embedded balance and strength training into habitual daily routines.	8/10	2++/C
Bian S, et al., 2018	Is there a relationship between milk consumption and the risk of hip fracture?	Systematic review and Meta-analysis	Consumption of yogurt and cheese was associated with lower risk of hip fracture in cohort studies. However, the consumption of total dairy products and cream was not significantly associated with the risk of hip fracture. There was insufficient evidence to deduce the association between milk consumption and risk of hip fracture. A lower threshold of 200 g/day milk intake may have beneficial effects, whereas the effects of a higher threshold of milk intake are unclear.	8/10	1+/B
Avenell A, et al., 2014.	What are the effects of vitamin D or related supplements, with or without calcium, on the prevention of hip fractures?	Systematic review	Vitamin D alone, in the formats and doses tested in older people, is unlikely to be effective in preventing hip fracture. There is high quality evidence that vitamin D plus calcium reduces the risk of any type of fracture	8/10	1+/B
Díaz Curiel M. 2015.	Does vitamin K prevent hip fractures?	Systematic review	There is currently insufficient evidence to recommend the routine use of vitamin K for the prevention of osteoporosis and fractures in postmenopausal women.	7/10	2++/C
Maximos M, et al., 2017.	Are antiepileptics related to hip fractures?	Systematic review	Health care professionals should monitor older adults while they take antiepileptic drugs to balance the need for such pharmacotherapy against an increased risk of falling and injuries from falls.	7/10	2++/C
Fernández-García M, et al., 2015.	What is the incidence of hip fracture in Spain?	Literature review	Total incidence of hip fracture in Spain among >65 year-olds has varied between 301 and 897/10^5^ inhabitants, values below those in other European countries or the U.S. Multidisciplinary treatment is fostered to prevent hip fractures.	7/10	3D
Pereira Rodrigues RM, et al., 2012.	What are the most effective guidelines for the prevention and treatment of glucocorticoid-induced osteoporosis?	Literature review	The minimum GC dose that indicates risk of fracture is 5 mg/day, the minimum GC use duration being 3 months. An adequate intake of calcium and vitamin D is important to prevent osteoporosis. However, Calcium carbonate at the dose of 1000 mg/day alone prevents neither bone mass loss nor fracture in patients initiating chronic GC use. Thus, it is not indicated for primary prevention. Modifiable risk factors, such as smoking, alcohol consumption (<3 daily units), sodium, sedentary lifestyle, and low weight should be removed or reduced. Resistive exercises with weight lifting and balance exercises are recommended.	9/10	2++/C
Fernández-García M, et al., 2015.	What is the incidence of hip fracture in Spain?	Literature review	Total incidence of hip fracture in Spain among >65 year-olds has varied between 301 and 897/105 inhabitants, values below those in other European countries or the U.S. Multidisciplinary treatment is fostered to prevent hip fractures.	7/10	2+/C
Moreira Fernandes DL, et al., 2014.	Multifactorial programme to prevent hip fracture in people older than 65 and postmenopausal women	Literature review	A combined exercise program (resistance + aerobic + impact) is recommended for an enhancement of spine BMD and exercises aimed at developing muscle strength and balance to prevent falls and fractures such as mechanical vibration, walking, running, water aerobics…	7/10	3/D

^1^ Critical Appraisal Skills Programme (CASPE) check list: Randomized controlled trial 11 items, Case control studies 11 items, cohort studies 12 items. ^2^ Scottish Intercollegiate Guidelines Network (SIGN).

**Table 3:** Results from clinical studies.

Authors Year	Research Question	Method	Results	CASP ^1^	SIGN ^2^
Shepstone L, et al., 2018.	Is a systematic detection programme based on communities at risk of fracture feasible?	Randomised controlled trial	Treatment was recommended in 898 (14%) of 6233 women. Use of osteoporosis medication was higher at the end of year 1 in the screening group compared with controls (15% vs 4%), with uptake particularly high (78% at 6 months) in the screening high-risk subgroup. Preliminary findings indicate that the cost per prevented osteoporotic-related fracture is less than £4500, and the cost per prevented hip fracture is less than £8000. Additionally, the cost per quality-adjusted life-year gained, estimated under various scenarios, was less than £20,000.	9/11	1+/B
McCloskey E, et al., 2018.	Utilization of FRAX	Randomised controlled trial	The analysis demonstrates an interaction between baseline FRAX hip fracture probability and a subsequent reduction in hip fracture incidence in those at higher risk targeted for appropriate treatment.	7/11	1+/B
Skelton DA, et al., 2016.	Does doing physical exercise decrease the risk falls in the visually impaired?	Randomised controlled trial	There are potential risks and benefits to introducing exercises to this population. FoF in VIOP may exacerbate existing gait and balance difficulties, further increasing the risk of falls.	7/11	1+/B
Turner DA, et al., 2018.	What is the economic impact of hip fractures?	Randomised controlled trial	A systematic study to detect community-based risk of fracture implies savings of £4478 and £7694 per fracture for osteoporosis-related and hip fractures, respectively.	7/11	1+/B
Fung TT, et al., 2017.	Does protein intake prevent firsk of falls?	Prospective cohort study	Among men, significant inverse associations were observed for each 10 g increase of total protein (RR = 0.92, 95% CI = 0.85–0.99) and animal protein (RR = 0.91, 95% CI = 0.85–0.98) intakes. Total and animal proteins were not significantly associated with hip fractures in women. Both plant (RR = 0.88, 95% CI 0.79–0.99 per 10 g) and dairy protein (RR = 0.92, 95% CI 0.86–0.97) were associated with significantly lower risks of hip fracture when results for men and women were combined.	8/12	2+/C
Hiligsmann M, et al., 2016.	Impact on public health and economic analysis of D-fortified diary products for fracture prevention	Cohort analytical observational study.	The total lifetime number of fractures was reduced by 64,932 for the recommended intake of dairy products in the general population over 65 years, of which 46,473 and 18,460 occurred in women and men, respectively. The cost per QALY gained was €58,244, in the range of commonly accepted threshold for cost-effectiveness. The intake of fortified dairy products becomes highly cost-effective (cost per QALY <€30,000) in women over 70 years and in men over 80 years.	7/12	2+/C
Leal J, et al., 2016.	What is the economic impact of a hip fracture?	Cohort study.	Hospital costs following hip fracture, estimated at £1.1 billion, are high and mostly occur in the first year after the index hip fracture. There is a strong economic incentive to prioritise research funds towards identifying the best approaches to prevent both index and subsequent hip fractures.	7/12	2+/C
Lee VW, et al., 2016.	Risk factors for falls in older people over 65 years?	Case control study	Risk factors for falls: Benign prostatic hyperplasia and lower urinary tract symptoms, First-generation antihistamines, antiparkinsonian medication, osteoporosis, and use of walking aids. Solution: Clinical medication revision, monitoring of medication-related adverse events and medication optimization.	8/11	2+/C
Brännström J, et al., 2019.	Is there an association between antidepressants and hip fracture?	Cohort study	Antidepressant users sustained more than twice as many hip fractures than did nonusers in the year before and year after the initiation of therapy (2.8% vs 1.1% and 3.5% vs 1.3%, respectively, per actual incidence figures).	8/12	2+/C
Holvik K, et al., 2015	Can high serum retinol concentrations counter vitamin D preventive effect?	Cohort and cases multicentre analysis	No evidence was found of an adverse effect of high serum retinol on hip fracture or any interaction between retinol and 25-hydroxyvitamin D. If anything, there tended to be an increased risk at low retinol concentrations. Cod liver oil should not be discouraged as a natural source of vitamin D supplementation for fracture prevention.	7/12	2+/C
Van Geel T, et al., 2018.	Impact of oral biphosphonate in preventing hip fractures	Cohort prospective	The recommended bisphosphonate treatment had a substantially lower risk of subsequent fragility fracture and lower risk of mortality.	7/12	2+/C
Søgaard AJ, et al., 2014.	Does obesity increase the risk of hip fracture?	Cohort study	The risk of hip fracture decreased with increasing body mass index, plateauing in obese men. However, higher waist circumference and higher waist-hip ratio were associated with an increased risk of hip fracture after adjustment for body mass index and other potential confounders. Women in the highest tertile of waist circumference had an 86% (95% CI: 51–129%) higher risk of hip fracture compared to the lowest, with a corresponding increased risk in men of 100% (95% CI 53–161%). Lower body mass index combined with abdominal obesity increased the risk of hip fracture considerably, particularly in men.	7/12	2+/C
Herland T, et al., 2016	Is airflow limitation associated with bone mineral density (BMD) and risk of hip fractures?	Cohort study	Smoking, low BMI, use of glucocorticoids, lack of exercise, and chronic airflow limitation foster hip fractures. Airflow limitation is associated with low BMD and the risk of hip fracture.	7/12	2+/C
Finnes TE, et al., 2015.	Are vitamin K1 and D related with a higher risk of hip fracture?	Case control study	A 50% higher risk of hip fracture was observed in subjects with both low vitamin K1 and 25(OH)D compared with subjects with high vitamin K1 and 25(OH)D. No increased risk was observed in the groups which presented low values in only one vitamin.	6/11	2+/C
Feskanich D, et al., 2014.	Is there an association between physical activity and the risk of hip fracture in men?	Cohort prospective study	The initial assessment in 1986 listed 8 activities: walking, jogging, running, bicycling, calisthenics or exercise machines, tennis, racquetball or squash, and lap swimming. Walking is a relatively safe and easy activity for hip fracture prevention.	7/12	2+/C
Øyen J, et al., 2014.	Is there a relationship between smoking and body fat mass in relation to bone mineral density and hip fracture?	Cohort study	The positive association between fat mass and BMD was stronger among moderate and heavy smokers compared to never smokers, and the relation was stronger in smokers with low fat mass or BMI compared with those who had them high.	10/12	2+/C
Rosendahl-Riise H, et al., 2018.	Effect of fish consumption on the risk of hip fracture.	Cohort study	During a mean (SD) follow-up time of 9.6 (2.7) years, 226 hip fractures (72 in men, 154 in women) were observed. The mean (SD) fish intake was 48 (25) g/1000 kcal. Low intake of fish is associated with an increased risk of hip fracture.	7/12	2+/C
Sánchez-Hernández N, et al., 2016.	How to manage geriatric patients with hip fracture	Cohort study	Higher rates of complications were recorded (delirium, malnutrition, anaemia, and electrolyte disorders).	7/12	2+/C
Olmo JA, et al., 2014.	Do patients with cerebrovascular accident have a risk of hip fracture?	Case control study	Twelve precent of those patients with CVA had an increased risk of suffering a hip fracture, and 8% an increased risk of a major osteoporotic fracture. A programme of physical therapy with weight and walking should be planned to prevent hip fractures.	7/11	2+/C
Johansen JS, et al., 2018.	How can medication safety in the elderly be improved?	Case control study	The effect of the intervention varies by number of medications at admission or discharge; age groups; patient responsibility for their own medication at discharge; length of hospital stay; referred from home, home-care or nursing home.	8/11	2+/C
Chalapud-Narváez LM, et al., 2017.	Does physical activity improve strength and balance in older people?	Quasi-experimental longitudinal study	For the implementation of physical activity programmes, sociodemographic and cultural characteristics of the population must be considered for a better adaptation of the exercises according to the context. Conclusion: Physical activity is effective in improving balance and muscle strength for lower limbs, and a suitable tool to preserve senior’s functionality and independence.	6/11	2+/C
Benetou V, et al., 2018.	Does Mediterranean diet prevent hip fractures?	Cohort study	Hip fracture risk was lower among men and women with moderate (HR 0.93; 95% CI 0.87–0.99) and high (HR 0.94; 95% CI 0.87–1.01) adherence to the score compared with those with low adherence.	7/12	2+/C
Machado-Duque ME, et al., 2017.	Is using benzodiazepines and opioids associated with risk of hip fractures?	Case control study	Using opioids (OR:4.49; 95%CI:2.72–7.42) and benzodiazepines (OR:3.73; 95%CI:1.60–8.70) in the month prior to the event was significantly associated with a greater probability of suffering a fall with hip fracture.	8/11	2+/C
Benetou V, et al., 2016.	Is fruit and vegetable intake associated with hip fracture?	Cohort study	Participants with intake of 1 or <1 servings/day of fruits and vegetables had 39% higher risk of hip fracture (pooled HR 1.39; 95% CI, 1.20 to 1.58), in comparison with those with moderate intake.	8/12	2+/C
Garcia Lopez M, et al., 2015.	The association between self-reported memory-loss and the risk of hip fracture.	Cohort study	There was a higher risk of hip fracture in elderly who reported self-perceived memory loss.	7/12	2+/C
Irwin AN; et al., 2015.	What is the economic impact of a hip fracture?	Retrospective cohort	The projected annual cost of osteoporosis care for 1000 women was $619,736 (CPOMS) versus $726,887 (comparator service).	6/12	2+/C
Tajeu GS, et al., 2013.	Does hip fracture affect death, debility, and destitution in people older than 65?	Cohort study	Hip fracture in elderly patients resulted in increased death, debility, and destitution. Initiatives that lead to improved treatment of osteoporosis could result in a decrease in incidence of fractures, subsequent death, debility, and destitution for older adults.	6/12	2+/C
Langsetmo L, et al., 2017.	Does protein intake influence hip fractures?	Cohort study	Higher total protein intake was also associated with a decreased risk of hip fracture (HR = 0.84 [95% CI, 0.73 to 0.95]).	6/12	2+/C
Giner M, et al., 2017.	Does obesity influence microstructure and biomechanical properties in patients with hip fracture?	Case control study	Obesity may be a protective factor of bone quality in the femoral region and has less effect on bone mineral density.	6/11	2+/C
Bakken MS, et al., 2016.	Does consuming antipsychotic drugs increase the incidence of hip fracture?	Cohort study	In people aged 60 and older in Norway, those who took an antipsychotic drug had twice the risk of sustaining a hip fracture.	6/12	2+/C
Dahl C, et al., 2015.	Is there a relation between water components and a decrease in hip fractures?	Case control study	A lower hip fracture risk in men (50–85 years) that were supplied water with relatively high calcium was found, and the association between calcium and hip fracture was stronger when also the copper level in the water was high.	6/11	2+/D
Bliemel C, et al., 2015.	Do Parkinson patients have a higher risk of hip fractures?	Cohort study	Patients with Parkinson’s Disease are at risk for specific complications and longer hospitalization at the time of transfer from acute care so as for reduced abilities in activities of daily living in the medium term.	6/12	2+/D
Lai CL, et al., 2015.	Do α-adrenoceptor blockers have an influence on the risk of hip fractures?	Self-controlled case series	Use of α-adrenoceptor blockers was associated with a small but significant increase in the risk of hip/femur fractures during the early initiation period in patients without concomitant prescriptions of antihypertensive agents.	6/11	3/D
Leland NE, et al., 2015.	What is the probability of suffering a hip fracture in patients during their stay?	Observational study	Only 14.6% of short-stay nursing home patients achieved successful community discharge; this emphasizes the devastating impact of a fall with injury during a SNF stay.	7/11	3/D

^1^ Critical Appraisal Skills Programme (CASPE) check list: Randomized controlled trial 11 items, Case control studies 11 items, cohort studies 12 items. ^2^ Scottish Intercollegiate Guidelines Network (SIGN).

**Table 4:** Results from descriptive studies.

Authors Year	Research Question	Method	Results	Quality ^1^	SIGN ^2^
Sosa Henríquez S.	Causes for osteoporosis and how to treat it?	Protocol	Osteoporosis is a silent process that hinders bone quality until, due to trauma or overload, bone micro or macroscopic fracture is produced and, thus, the bone fractures. Causes for osteoporosis: Endocrinopathies, digestive diseases, haematological disorders, connective tissue diseases, drugs, nutritional alterations, immobilisation…	High	1+/B
Dore DD, et al., 2018.	Does nonbenzodiazepine hypnotics use influence hip fracture rates?	Case-crossover study	Doses of these prescribed hypnotic drugs should be kept as low as possible, especially among those with advanced age, as the rate of hip fracture in NH residents due to use of non-benzodiazepine hypnotics was greater among older patients than among younger patients.	Medium	2+/C
Visschedijk J, et al., 2014.	Which factors explain fear of falling?	Cross-sectional descriptive study	Multivariate analysis showed that walking ability before fracture (odds ratio (OR) 0.34, 95% confidence interval (CI) 0.14–0.83), activities of daily living after fracture (OR 0.89, 95% CI 0.80–0.99), and anxiety (OR 1.22, 95% CI 1.05–1.42) were independently associated with fear of falling.	Medium	2+/C
Gómez Navarro R, et al., 2017.	Can fragility fracture be prevented?	Cross-sectional descriptive study	Osteoporosis can and should be prevented, diagnosed and treated, preferably before occurrence of fragility fractures.	High	3+/D
Amador-Licona N, et al., 2018.	In patients with hip fracture, does protein and serum lipids intake influence muscle strength?	Cross-sectional descriptive study	The elderly, predominantly women and with a homogeneous age of 80 years on average, were malnourished or at risk of malnutrition in 93% of cases according to the MNA. Men consumed significantly more protein than women. Muscle strength is negatively associated with triglyceride levels; 36% of the elderly had triglyceride levels above 150 mg/dl. The daily protein recommendation for adults is 0.8 g/kg/day; however, several studies argue that an intake of 1.0–1.5 g protein/kg/day could benefit the health of the elderly.	Medium	3/D
Guimarães Rodriguez I, et al., 2014.	Segments of the elderly population with higher likelihood of suffering falls through the identification of associated factors.	Cross-sectional study	Falls were more frequent, after adjustment for gender and age, among female elderly participants; elderly adults (80 years old and older); widowed; and among elderly adults who had rheumatism/arthritis/arthrosis, osteoporosis, asthma/bronchitis/emphysema, headache, mental common disorder, dizziness, insomnia, use of multiple medications (five or more), and use of cane/walker.	Medium	3/D
León Vázquez F, et al., 2015.	Prevention of osteoporotic fracture in Spain: use of drugs before and after a hip fracture	Cross-sectional study.	Before the fracture, 26.5% (95% confidence interval [CI]: 24.8–28.1%) had received some antiosteoporotic treatment, of which 12% (95% CI: 11.0–13.5%) were bisphosphonates; 38.6% (95%CI: 36.8–40.4%) received treatment after the fracture, 20.4% (95%: 18.9–22%) were treated with bisphosphonates. Hip fracture produces the most mortality and morbidity. To start the treatment to prevent hip fractures, the possible gastric pathologies must be analysed, as they act as contraindications for bisphosphonates intake, apart from other clinical characteristics such as hyperthyroidism. Diabetes mellitus type 2, malabsorption syndrome, malnutrition, male hypogonadism, early menopause, rheumatoid arthritis and osteoporosis, and the previous use of drugs such as corticoids. In primary prevention, special emphasis shall be put on modifiable risk factors, as the effectiveness of drugs is controversial and, in the case of benefit, there would be little. There is a consensus that population screening regarding BMD with densitometry shall not be recommended, but only applied in cases of high risk and in decision-making regarding relevant therapeutic decisions.	High	3+/D
Montoya MJ, et al., 2017.	Does vitamin D influence the bone structure in patients with hip fracture?	Cross-sectional observational study	Vitamin D levels are low in the elderly, mostly in those patients with osteoporotic hip fracture. In the sample, there are high levels of PTH and bone remodelling markers, and lower levels of BMD, which indicates a higher probability of hip fracture.	Medium	3/D
WHO	How to prevent osteoporotic hip fracture	Experts opinion	Physical activity that maintains or increases muscle strength, coordination, and balance is beneficial towards preventing osteoporotic fractures. In those countries where the incidence of osteoporotic fractures is high, reduced calcium intake (that is, lower than 400–500 mg/day) is associated with higher risk of fracture among both men and women.	High	4/D
Soliman Y, et al., 2016.	Is urinary incontinence a risk factor of falls?	Experts opinion	Many falls are associated with the physical condition of a person or a medical condition such as arthritis, benign prostatic hyperplasia (BPH) or overactive bladder (OAB). Other causes include certain drugs and lower urinary tract symptoms. Both BHP and OAB are associated with nocturia, which commonly is the predominant factor for falls and fractures at night.	Medium	4/D
IOF	What are the needed calcium amounts to prevent hip fractures?	Experts opinion	With age, the body’s ability to absorb calcium declines, which is one of the reasons why seniors also require higher amounts. Milk and dairy products are the most readily available dietary sources of calcium. Caffeine and salt can increase calcium loss from the body and should not be taken in excessive amounts. Alcohol should also be taken in moderation as it detracts from bone health and is associated with falls and fractures. No conclusive evidence shows that fizzy soft drinks weaken bones.	High	4/D

^1^ Berra S. et al. tool for descriptive studies [37]. ^2^ Scottish Intercollegiate Guidelines Network (SIGN).

**Table 5:** Results from clinical practice guideline.

Authors	Research Question	Method	Results	AGREE ^1^	SIGN ^2^
NICE	Are there any possible treatments apart from pharmacological?	Clinical Practice Guideline	Bisphosphonates are more effective than placebo in reducing the risk of fractures. The risk assessment tools used in clinical practice are FRAX and QFracture.	Recommended	1+/B
NICE	Is it important to identify the risk factors to prevent hip fractures?	Clinical Practice Guideline	Projections suggest that, in the UK, hip fracture incidence will rise from 70,000 per year in 2006 to 91,500 in 2015 and 101,000 in 2020.	Recommended	1+/B
NICE	Do people who have had a fragility fracture or who take systemic glucocorticoids or who have a history of falls have a greater risk of fractures?	Clinical Practice Guideline	Maintain treatment if T score is lower than −2.5 and reassess the risk of fracture and bone mineral density every 3 to 5 years	Recommended	1+/B

^1^ Appraisal of Guidelines Research and Evaluation. ^2^ Scottish Intercollegiate Guidelines Network (SIGN).

**Table 6:** Recommended interventions related to the Nursing Interventions Classification.

Risk Factor	Recommended Intervention	SIGN ^1^	NIC ^2^
Nutritional deficit	- Train the patient in specific dietary needs according to their development or age (increased intake of calcium and vitamin D) - Abandon toxic habits or, if not possible, decrease them as much as possible (alcohol and caffeine) - Determine the nutritional status of the patient and their ability to meet their nutritional needs	2++/B	1100 Nutrition management
	- Ca and vitamin D supplements in patients treated with glucocorticoids, that consume isoflavones, and with an intake of the following supplements: magnesium, copper, zinc, iron, silica, boron, strontium, manganese, and over-protein consumption	1+/C	
Orthostatic hypotension	- Posture recommendations: get up slowly twice (first sit down, and then get up), raise the bedhead for a while before getting up, always use bearing position. - Decrease the dose or withdraw or replace those drugs that may cause hypotension. - Measurement of resting blood pressure. - Perform 12-lead electrocardiogram if require.	2+/B	6490 Fall prevention
Use of benzodiazepines or other sedatives, antidepressants, antipsychotics, H1 antihistamines Polymedicated	- Avoid the use of dubious or unproven drugs - Try to decrease doses, if possible; educate on the correct use of sedatives and hypnotics (avoid alcohol, interactions with other drugs, seek proper compliance). - Avoid, whenever possible, the use of osteopenic drugs (oral glucocorticoids, l-thyroxine, heparin, antiepileptics, neuroleptics, chemotherapy...) - Periodically check with the patient and/or family the types and doses of medicines taken.	2++/B	2380 Medication management
Dangerous environment Dependent for daily activities	- Well-lit rooms and environment. - Be cautious with pets. - Notify the authorised institutions to protect the environment (Ministry of Health, Environmental Services, Environmental Protection Agency, and Police Forces). - Avoid loose carpets. - Use protective devices (physical restraints, railings, closed doors, fences, and gates) to physically limit mobility or access to hazardous situations. - Beware of wet floor and shower trays. - Identify internal and external factors that may improve or decrease motivation to adhere to healthy behaviours. - Encourage the autonomy of the patient as much as possible. - Tips on non-pharmacological measures for sleep problems (avoiding daytime sleep, exercising before bed, taking hot drinks, applying relaxation techniques). - Tidy rooms; do not leave objects on the floor.	2+/B	1850 Sleep enhancement 6486 Environmental management: safety 5510 Health education
Difficulties for transfers (bed- chair, chair-WC)	- Modifications of the environment (chairs with armrests, handles in toilet and room, raise toilet seat, evaluate the height of the bed, railings...) - Train both the patient and the family and/or caregivers on the appropriate techniques for moving from one area to another (from bed to chair, from wheelchair to vehicle) - At the end of the transfer, assess the patient’s proper alignment of the body, that probes are not occluded, wrinkle-free bedding, unnecessarily exposed skin, appropriate level of comfort of the patient, and side railings of the raised bed	2+/B	1806 Self-care assistance: transfer
Difficulties for walking and/or balance	- Physiotherapy if needed - Water exercises	2++/B	0221Excercise therapy: ambulation
	- Apply/provide aid devices (cane, crutches or wheelchair...) for wandering if the patient has instability	2+/C	
	- Muscle-boosting exercises (psoas, quadriceps) - Scheduled walking exercises (indoor and outdoor) if possible (15 min. twice a day) - Exercises on vibrating platforms (frequency 25 to 35 Hz) 6 min per session and twice a week to improve balance	LE:2++ DR:B	0200 Exercise promotion
Impaired vision	- Monitor the functional implications of decreased vision (e.g., risk of injury, depression, anxiety and ability to perform daily life activities, as well as valued activities) - Ensure that the patient’s glasses or lenses have an up-to-date prescription, are clean and properly stored when not in use - Treatment of ophthalmological diseases if applicable (cataracts, glaucoma, diabetic retinopathy...)	2+/B	4978 Communication enhancement visual deficit
Cognitive impairment	- Assessment of cognitive impairment and/or existence of delirium, and applying environmental corrective measures - Assess the existence of hidden depression and treat it if appropriate - Psychostimulation measures, avoidance of sedatives	2+/B	4720 Cognitive stimulation 6460 Dementia management
Use of urinary incontinence absorbers	- Identify multifactorial causes for incontinence (diuresis, voiding pattern, cognitive function, previous urinary problems, post-void residual urine, and medications) - Limit the use of absorbers to the maximum or look for other solutions to the problem of incontinence	2+/B	0610 Urinary incontinence care

^1^ Scottish Intercollegiate Guidelines Network (SIGN). ^2^ Nursing Interventions Classification.

## References

[1] Osteoporosis: Assessing the Risk of Fragility Fracture. Clinical Guideline. NICE. https://www.nice.org.uk/guidance/cg146.

[2] Shepstone L., Lenaghan E., Cooper C., Clarke S., Fong-Soe-Khioe R., Fordham R., Gittoes N., Harvey I., Harvey N., Heawood A. (2018). Screening in the community to reduce fractures in older women (SCOOP): A randomised controlled trial. Lancet.

[3] Turner D., Khioe R.F.S., Shepstone L., Lenaghan E., Cooper C., Gittoes N., Harvey N., Holland R., Howe A., McCloskey E. (2018). The Cost-Effectiveness of Screening in the Community to Reduce Osteoporotic Fractures in Older Women in the UK: Economic Evaluation of the SCOOP Study. J. Bone Miner. Res..

[4] Tajeu G.S., Delzell E., Smith W., Arora T., Curtis J.R., Saag K.G., Morrisey M.A., Yun H., Kilgore M.L. (2013). Death, Debility, and Destitution Following Hip Fracture. J. Gerontol. Ser. A Biol. Sci. Med. Sci..

[5] Irwin A.N., Billups S.J., Heilmann R.M.F. (2015). Labor Costs and Economic Impact of a Primary Care Clinical Pharmacy Service on Postfracture Care in Postmenopausal Women. Pharmacother. J. Hum. Pharmacol. Drug Ther..

[6] Osteoporosis and Musculoskeletal Disorders. Osteoporosis. Calcium IOF. https://www.iofbonehealth.org/osteoporosis-musculoskeletal-disorders/osteoporosis/prevention/calcium.

[7] Ballesteros-Álvaro A.M., Crespo-de las Heras M.I., Pérez-Alonso J., Delgado-González E., González-Esteban M.P. (2013). Intervenciones No Farmacológicas Que son Efectivas para Prevenir la Fractura de Cadera por Fragilidad en Atención Primaria. Evidentia.

[8] Joint WHO/FAO Expert Consultation (2003). Dieta, Nutrición y Prevención de Enfermedades Crónicas.

[9] Osteoporosis and Musculoskeletal Disorders. Osteoporosis. Fracture Risk Assessment. IOF. https://www.iofbonehealth.org/osteoporosis-musculoskeletal-disorders/osteoporosis/diagnosis/fracture-risk-assessment.

[10] Rapp K., Büchele G., Dreinhöfer K., Bücking B., Becker C., Benzinger P. Epidemiology of Hip Fractures. Zeitschrift für Gerontologie und Geriatrie.

[11] Fernández-García M., Martínez J., Olmos J., González-Macías J., Hernández J. (2015). Tendencia secular de la incidencia de la fractura de cadera en el mundo. Rev. Osteoporos. Metab. Miner..

[12] Crisp A., Dixon T., Jones G., Cumming R.G., Laslett L.L., Bhatia K., Webster A., Ebeling P.R. (2012). Declining incidence of osteoporotic hip fracture in Australia. Arch. Osteoporos..

[13] Fernández-García M., Martínez J., Olmos J., González-Macías J., Hernández J. (2015). Revisión de la incidencia de la fractura de cadera en España. Rev. Osteoporos. Metab. Miner..

[14] Carbonell Abella C., Martinez Laguna D., Muñoz Torres M., Nogués Solán X., Perez Martin A. (2013). Fragilidad Ósea.

[15] Søgaard A.J., Holvik K., Omsland T.K., Tell G.S., Dahl C., Schei B., Falch J.A., Eisman J.A., Meyer H.E. (2014). Abdominal obesity increases the risk of hip fracture. A population-based study of 43,000 women and men aged 60–79 years followed for 8 years. Cohort of Norway. J. Intern. Med..

[16] Giner M., Montoya-García M.-J., Miranda C., Vázquez M.Á., Miranda M., Pérez-Cano R. (2017). Influencia de la obesidad sobre la microarquitectura y las propiedades biomecánicas en pacientes con fractura de cadera. Rev. Osteoporos. Metab. Miner..

[17] Lopez M.G., Omsland T.K., Søgaard A.J., Meyer H.E. (2015). Self-perceived memory loss is associated with an increased risk of hip fracture in the elderly: A population-based NOREPOS cohort study. BMC Geriatr..

[18] Osteoporosis. Clinical Guideline. NICE. https://www.nice.org.uk/guidance/qs149..

[19] Bakken M.S., Schjøtt J., Engeland A., Engesaeter L.B., Ruths S., Engesæter L.B. (2016). Antipsychotic Drugs and Risk of Hip Fracture in People Aged 60 and Older in Norway. J. Am. Geriatr. Soc..

[20] Soliman Y., Meyer R., Baum N. (2016). Falls in the Elderly Secondary to Urinary Symptoms. Rev. Urol..

[21] Visschedijk J., Caljouw M., Balen R., Hertogh C., Achterberg W. (2014). Fear of falling after hip fracture in vulnerable older persons rehabilitating in a skilled nursing facility. J. Rehabil. Med..

[22] Rodrigues I.G., Fraga G.P., Barros M.B.D.A. (2014). Falls among the elderly: Risk factors in a population-based study. Rev. Bras. Epidemiol..

[23] Benetou V., Orfanos P., Feskanich D., Michaëlsson K., Pettersson-Kymmer U., Eriksson S., Grodstein F., Wolk A., Bellavia A., Ahmed L.A. (2016). Fruit and Vegetable Intake and Hip Fracture Incidence in Older Men and Women: The CHANCES Project. J. Bone Miner. Res..

[24] Bliemel C., Oberkircher L., Eschbach D., Lechler P., Balzer-Geldsetzer M., Ruchholtz S., Bücking B. (2015). Impact of Parkinson’s disease on the acute care treatment and medium-term functional outcome in geriatric hip fracture patients. Arch. Orthop. Trauma Surg..

[25] Leland N.E., Gozalo P., Bynum J., Mor V., Christian T.J., Teno J.M. (2015). What happens to patients when they fracture their hip during a skilled nursing facility stay?. J. Am. Med. Dir. Assoc..

[26] Chen K., Chang S., Lin P. (2017). Frailty as a Predictor of Future Fracture in Older Adults: A Systematic Review and Meta-Analysis. Worldviews Evid.-Based Nurs..

[27] McCloskey E., Johansson H., Harvey N., Shepstone L., Lenaghan E., Fordham R., Harvey I., Howe A., Cooper C., Clarke S. (2018). Management of Patients with High Baseline Hip Fracture Risk by FRAX Reduces Hip Fractures-A Post Hoc Analysis of the SCOOP Study. J. Bone Miner. Res..

[28] Navarro R.G., García P.G., Hernández C.M., Sauras Á.C., Enguídanos S.V. (2017). [Primary and Secondary Prevention of Hip Fragility Fracture in Teruel Health Sector, Aragon, Spain]. Rev. Esp. Salud Publica.

[29] Arksey H., O’Malley L. (2005). Scoping studies: Towards a methodological framework. Int. J. Soc. Res. Methodol..

[30] Moher D., Liberati A., Tetzlaff J., Altman U.G. (2009). Preferred Reporting Items for Systematic Reviews and Meta-Analyses: The PRISMA Statement. PLoS Med..

[31] Alonso Coello P., Ezquerro Rodríguez O., Fargues Garcia I., Garcia Alamino J.M., Marzo Castillejo M., Navarra Llorens M., Pardo Pardo J., Subirana Casacuberta M., Urrutia Cuchí G. (2004). Enfermería Basada en la Evidencia. Hacia la Excelencia en los Cuidados.

[32] (2018). CASPe. Programa de Habilidades en Lectura Crítica Español. Alicante (España). http://www.redcaspe.org/herramientas/instrumentos.

[33] Berra S., Elorza-Ricart J.M., Estrada M.-D., Sánchez E. (2008). Instrumento para la lectura crítica y la evaluación de estudios epidemiológicos transversales. Gac. Sanit..

[34] Flórez Gómez I.D., Montoya D.C. (2011). Las guías de práctica clínica y el instrumento AGREE II. Metodología de investigación y lectura crítica de estudios. Rev. Colomb. de Psiquiatr..

[35] Marzo–Castillejo M., Viana-Zulaica C. (2007). Calidad de la evidencia y grado de recomendación. Guías Clín..

[36] Holvik K., Ahmed L.A., Forsmo S., Gjesdal C.G., Grimnes G., Samuelsen S.O., Schei B., Blomhoff R., Tell G.S., Meyer H.E. (2015). No increase in risk of hip fracture at high serum retinol concentrations in community-dwelling older Norwegians: The Norwegian Epidemiologic Osteoporosis Studies. Am. J. Clin. Nutr..

[37] Dahl C., Søgaard A.J., Tell G.S., Forsén L., Flaten T.P., Hongve D., Omsland T.K., Holvik K., Meyer H.E., Aamodt G. (2015). Population data on calcium in drinking water and hip fracture: An association may depend on other minerals in water. A NOREPOS 1 1Norwegian Epidemiologic Osteoporosis Studies. study. Bone.

[38] Bian S., Hu J., Zhang K., Wang Y., Yu M., Ma J. (2018). Dairy product consumption and risk of hip fracture: A systematic review and meta-analysis. BMC Public Health.

[39] Avenell A., Mak J.C.S., O’Connell D. (2014). Vitamin D and vitamin D analogues for preventing fractures in post-menopausal women and older men. Cochrane Database Syst. Rev..

[40] Finnes T., Lofthus C.M., Meyer H.E., Søgaard A.J., Tell G.S., Apalset E.M., Gjesdal C., Grimnes G., Schei B., Blomhoff R. (2015). A combination of low serum concentrations of vitamins K1 and D is associated with increased risk of hip fractures in elderly Norwegians: A NOREPOS study. Osteoporos. Int..

[41] Hiligsmann M., Burlet N., Fardellone P., Al-Daghri N., Reginster J.Y. (2016). Public health impact and economic evaluation of vitamin D-fortified dairy products for fracture prevention in France. Osteporos. Int..

[42] (2017). Vitamin D—International Osteoporosis Foundation. https://www.iofbonehealth.org/osteoporosis-musculoskeletal-disorders/osteoporosis/prevention/vitamin-d.

[43] (2017). Recomendaciones Sobre Osteoporosis. Sociedad Española de Reumatología. https://www.ser.es/wp-content/uploads/2018/03/Recomendaciones_OP_DEF.pdf.

[44] Weaver C.M., Alexander D.D., Boushey C.J., Dawson-Hughes B., Lappe J.M., LeBoff M.S., Liu S., Looker A.C., Wallace T.C., Wang D.D. (2015). Calcium plus vitamin D supplementation and risk of fractures: An updated meta-analysis from the National Osteoporosis Foundation. Osteoporos. Int..

[45] Montoya M.J., Vázquez M.A., Miranda C., Miranda M.J., Pérez-Cano R., Giner M. (2017). Influencia de la vitamina D sobre la microestructura y propiedades biomecánicas de pacientes con fractura de cadera. Rev. Osteoporos. Metab. Miner..

[46] Poole C.D., Smith J.C., Davies J.S. (2014). The short-term impact of vitamin D-based hip fracture prevention in older adults in the United Kingdom. J. Endocrinol. Investig..

[47] Amador-Licona N., Moreno-Vargas E.V., Martínez-Cordero C. (2018). Ingesta de Proteína, Lípidos Séricos y Fuerza Muscular en Ancianos. Nutr. Hosp..

[48] Fung T.T., Meyer H.E., Willett W.C., Feskanich D. (2017). Protein intake and risk of hip fractures in postmenopausal women and men age 50 and older. Osteoporos. Int..

[49] Langsetmo L., Shikany J.M., Cawthon P.M., Cauley J.A., Taylor B.C., Vo T.N., Bauer U.C., Orwoll E.S., Schousboe J.T., Ensrud K.E. (2017). The Association Between Protein Intake by Source and Osteoporotic Fracture in Older Men: A Prospective Cohort Study. J. Bone Miner. Res..

[50] Díaz Curiel M. (2015). Action of vitamin K on bone health. Rev. Osteoporos. Metab. Miner..

[51] Rosendahl-Riise H., Sulo G., Karlsson T., Drevon C.A., Dierkes J., Tell G.S. (2018). Limited Benefit of Fish Consumption on Risk of Hip Fracture among Men in the Community-Based Hordaland Health Study. Nutrition.

[52] Øyen J., Gjesdal C.G., Nygård O.K., Lie S.A., Meyer H.E., Apalset E.M., Ueland P.M., Pedersen E.R., Midttun Ø., Vollset S.E. (2014). Smoking and Body Fat Mass in Relation to Bone Mineral Density and Hip Fracture: The Hordaland Health Study. PLoS ONE.

[53] Herland T., Apalset E.M., Tell G.S., Lehmann S., Eide G.E. (2016). Airflow limitation as a risk factor for low bone mineral density and hip fracture. Eur. Clin. Respir. J..

[54] Pereira R., De Carvalho J.F., Paula A.P., Zerbini C., Domiciano D.S., Gonçalves H., Danowski J.S., Neto J.F.M., Mendonça L.M.C., Bezerra M.C. (2012). Guidelines for the prevention and treatment of glucocorticoid-induced osteoporosis. Rev. Bras. Reum..

[55] Bisphosphonates for Treating Osteoporosis. NICE. https://www.nice.org.uk/guidance/ta464.

[56] Narváez L.M.C., Almario A.E.E. (2017). Actividad física para mejorar fuerza y equilibrio en el adulto mayor. Univ. Salud.

[57] Feskanich D., Flint A.J., Willett W.C. (2014). Physical activity and inactivity and risk of hip fractures in men. Am. J. Public Health.

[58] Skelton D.A., Bailey C., Howel D., Cattan M., Deary V., Coe D., De Jong L.D., Gawler S., Gray J., Lampitt R. (2016). Visually Impaired OLder people’s Exercise programme for falls prevenTion (VIOLET): A feasibility study protocol. BMJ Open.

[59] Machado-Duque M.E., Castaño-Montoya J.P., Medina-Morales D.A., Castro-Rodríguez A., González-Montoya A., Machado-Alba J.E. (2017). Association between the use of benzodiazepines and opioids with the risk of falls and hip fractures in older adults. Int. Psychogeriatr..

[60] Brännström J., Lövheim H., Gustafson Y., Nordström P. (2019). Association Between Antidepressant Drug Use and Hip Fracture in Older People Before and After Treatment Initiation. JAMA Psychiatry.

[61] Lai C.-L., Kuo R.N., Chen H.-M., Chen M.-F., Chan K.A., Lai M.-S. (2015). Risk of hip/femur fractures during the initiation period of α-adrenoceptor blocker therapy among elderly males: A self-controlled case series study. Br. J. Clin. Pharmacol..

[62] Dore D., Zullo A.R., Mor V., Lee Y., Berry S.D. (2018). Age, Sex, and Dose Effects of Nonbenzodiazepine Hypnotics on Hip Fracture in Nursing Home Residents. J. Am. Med. Dir. Assoc..

[63] Sosa-Henríquez M., Curiel M.D., Pérez A.D., Alonso C.G., Macías J.G., Minguella J.F., Rubio J.F., Saidler L.M., Solán X.N., Hernandez D.H. (2008). Guía de prevención y tratamiento de la osteoporosis inducida por glucocorticoides de la Sociedad Española de Medicina Interna. Rev. Clín. Esp..

[64] Johansen J.S., Havnes K., Halvorsen K.H., Haustreis S., Skaue L.W., Kamycheva E., Mathiesen L., Viktil K.K., Granas A.G., Garcia B.H. (2018). Interdisciplinary collaboration across secondary and primary care to improve medication safety in the elderly (IMMENSE study): Study protocol for a randomised controlled trial. BMJ Open.

[65] Vázquez F.L., Bonis J., Cerezo V.B., Hernández S.H., Sánchez L.J., Holgado Diaz A. (2015). Prevención de fractura osteoporótica en España: Uso de fármacos antes y después de una fractura de cadera. Rev. de Osteoporos. Metab. Miner..

[66] Lee V.W.Y., Leung T.P.Y., Lee V.W.H. (2016). Outpatient Medication Use in Chinese Geriatric Patients Admitted for Falls. Am. J. Ther..

[67] Van Geel T.A.C.M., Bliuc D., Geusens P.P.M., Center J.R., Dinant G.-J., Tran T., van den Bergh J., McLellan A.R., Eisman J.A. (2018). Reduced mortality and subsequent fracture risk associated with oral bisphosphonate recommendation in a fracture liaison service setting: A prospective cohort study. PLoS ONE.

[68] Olmo J., Roman P., Leon M., Mena P., Ignatowitz U., Fuentes M., Almagro M., Martínez E., Torres J., Canteras M. (2014). Riesgo de fractura osteoporótica mayor y de cadera en pacientes con accidente cerebrovascular en fase aguda: Estudio prospectivo multicéntrico. Rev. Osteoporos. Metab. Miner..

[69] Sánchez-Hernández N., Sáez-López P., Paniagua-Tejo S., Valverde-García J. (2016). Resultados tras la aplicación de una vía clínica en el proceso de atención al paciente geriátrico con fractura de cadera osteoporótica en un hospital de segundo nivel. Rev. Esp. Cir. Ortop. Traumatol..

[70] Tiedemann A., Sherrington C., Lord S.R. (2013). The role of exercise for fall prevention in older age. Motriz Rev. Educ. Fís..

[71] Fernandez M.A., Griffin X.L., Costa M.L. (2015). Management of hip fracture: Figure 1. Br. Med. Bull..

[72] Maximos M., Chang F., Patel T. (2017). Risk of falls associated with antiepileptic drug use in ambulatory elderly populations. Can. Pharm. J. Rev. Pharm. Can..

[73] Cianferotti L., Fossi C., Brandi M.L. (2015). Hip Protectors: Are They Worth it?. Calcif. Tissue Int..

[74] Santesso N., Carrasco-Labra A., Brignardello-Petersen R. (2014). Hip protectors for preventing hip fractures in older people. Cochrane Database Syst. Rev..

[75] Romero Pérez A., Rivas Velasco A. (2014). Adherence to Mediterranean diet and bone health. Nutr. Hosp..

